# Supplementary figures and images for: Beyond the transcript: Chromatin implications in trans-splicing in Trypanosomatids
Source: PLoS One. 2026 Feb 26;21(2):e0343367. doi: 10.1371/journal.pone.0343367 (PMC12944795; doi:10.1371/journal.pone.0343367)

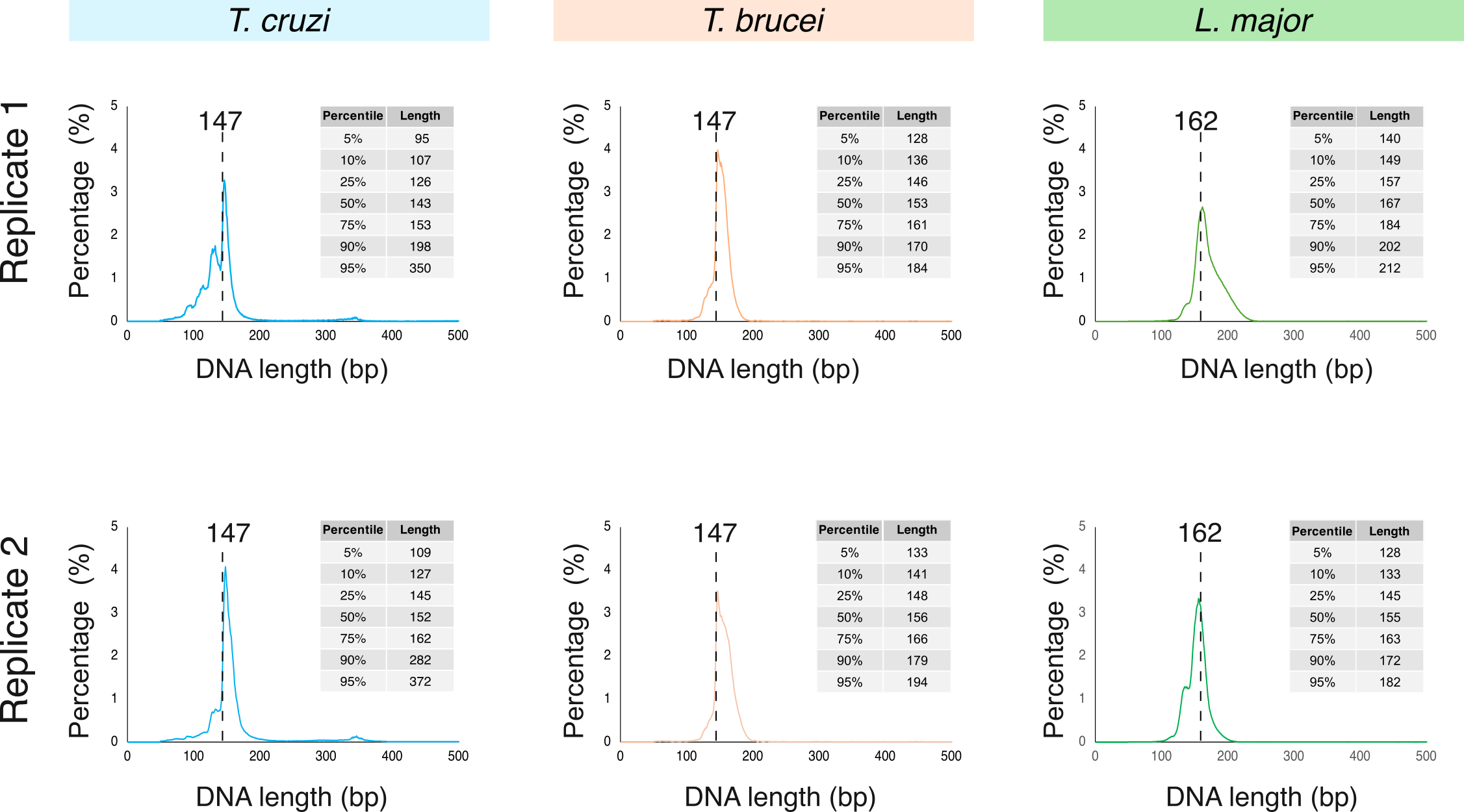

Supplement: S1 Fig — Length histogram for all nucleosomal DNA sequenced for two replicated experiments for T. cruzi CL Brener replicate 1 (GSM5363006) and replicate 2 (GSM5363007) (left panels), T. brucei 427 replicate 1 (GSM2407366) and replicate 2 (GSM2407367) (middle panels), and L. major Friedlin replicate 1(GSM2179742) and replicate 2 (GSM2179741) (right panels) respectively. Dashed lines indicate the length of the more abundant DNA fragments in the sample. (TIFF) [file pone.0343367.s001.tiff]

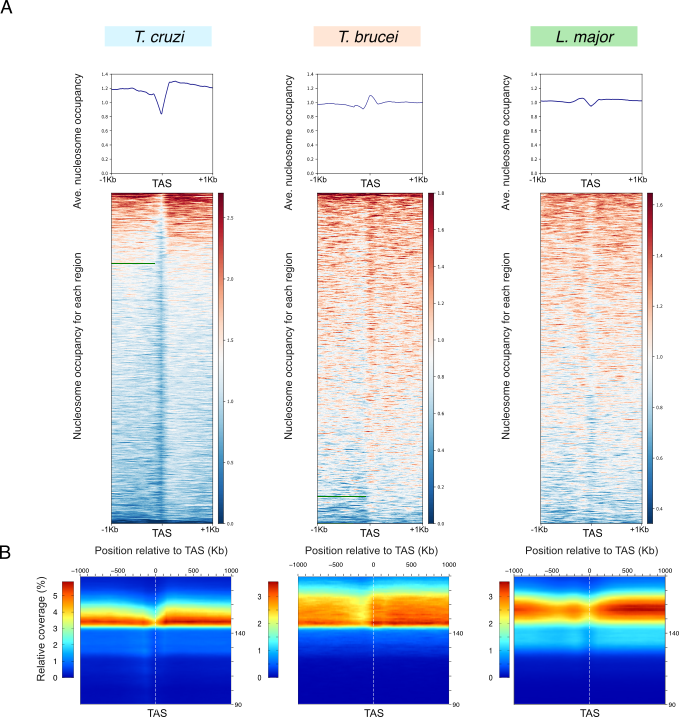

Supplement: S2 Fig — (A)Average nucleosome occupancy (top panels), heatmaps for each region in a 1 kb window (bottom panels). The signals scored for DNA molecules in the nucleosomal-size range (120–180 bp) are represented; (B) 2D occupancy plots showing nucleosome density relative to the TAS for all the sequenced DNA for a replicate experiment of T. cruzi (GSM5363007), T. brucei (GSM2407367) and L. major (GSM2179741) respectively. Red: High nucleosome density; blue: low nucleosome density. (TIFF) [file pone.0343367.s002.tiff]

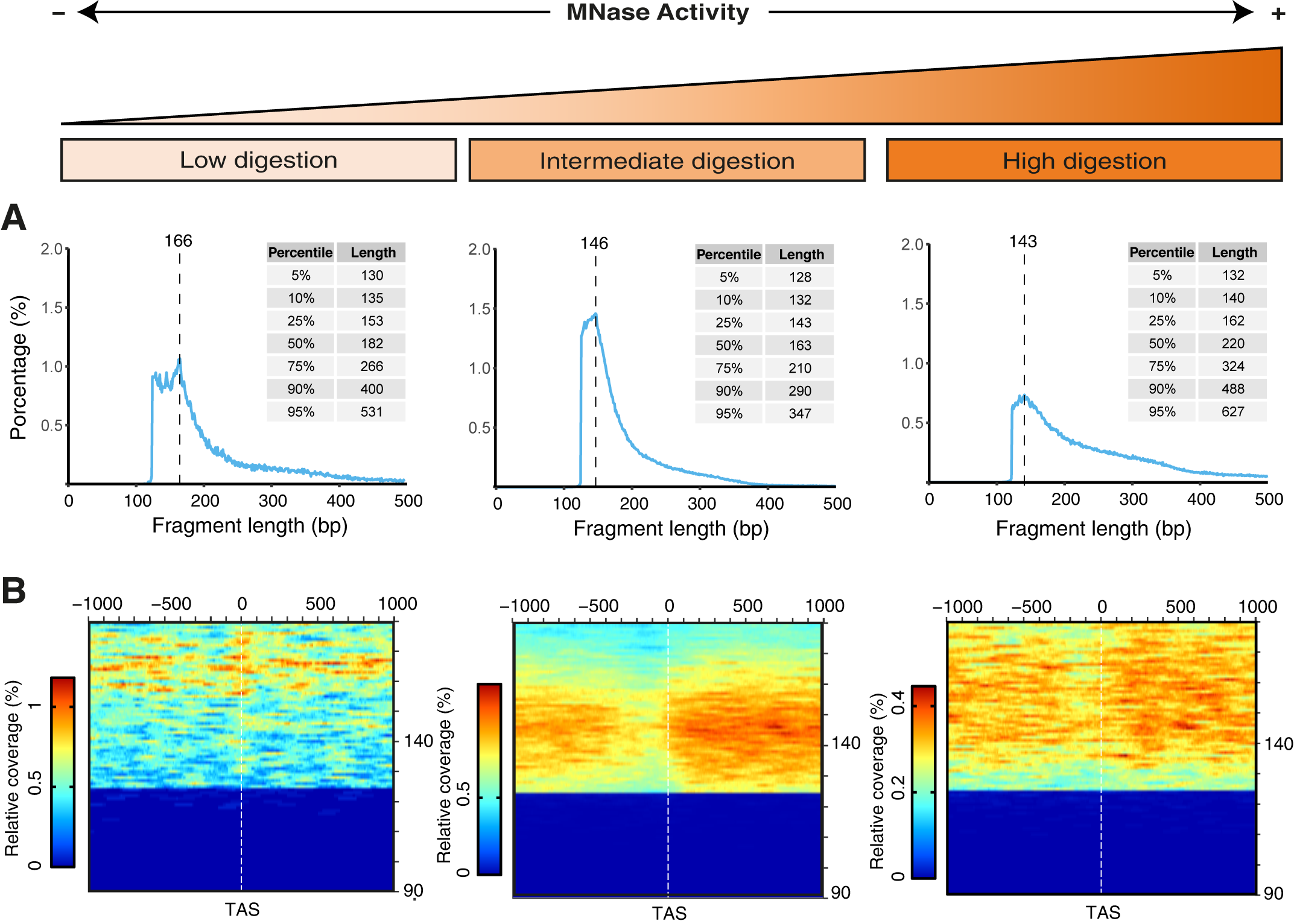

Supplement: S3 Fig — (A) Length distribution histogram for sequenced DNA molecules for T. brucei 427 samples with different extent of MNase digestions. Each data set corresponds to the ones represented in Fig 2. (B) 2D occupancy plots. Red: High nucleosome density; blue: low nucleosome density. Dashed lines indicate the length of the more abundant DNA fragments in the sample. The data sets used in this figure are: Low digestion (GSM5024927), intermediate digestion (GSM5024915) and high digestion (GSM5024921). (TIF) [file pone.0343367.s003.tif]

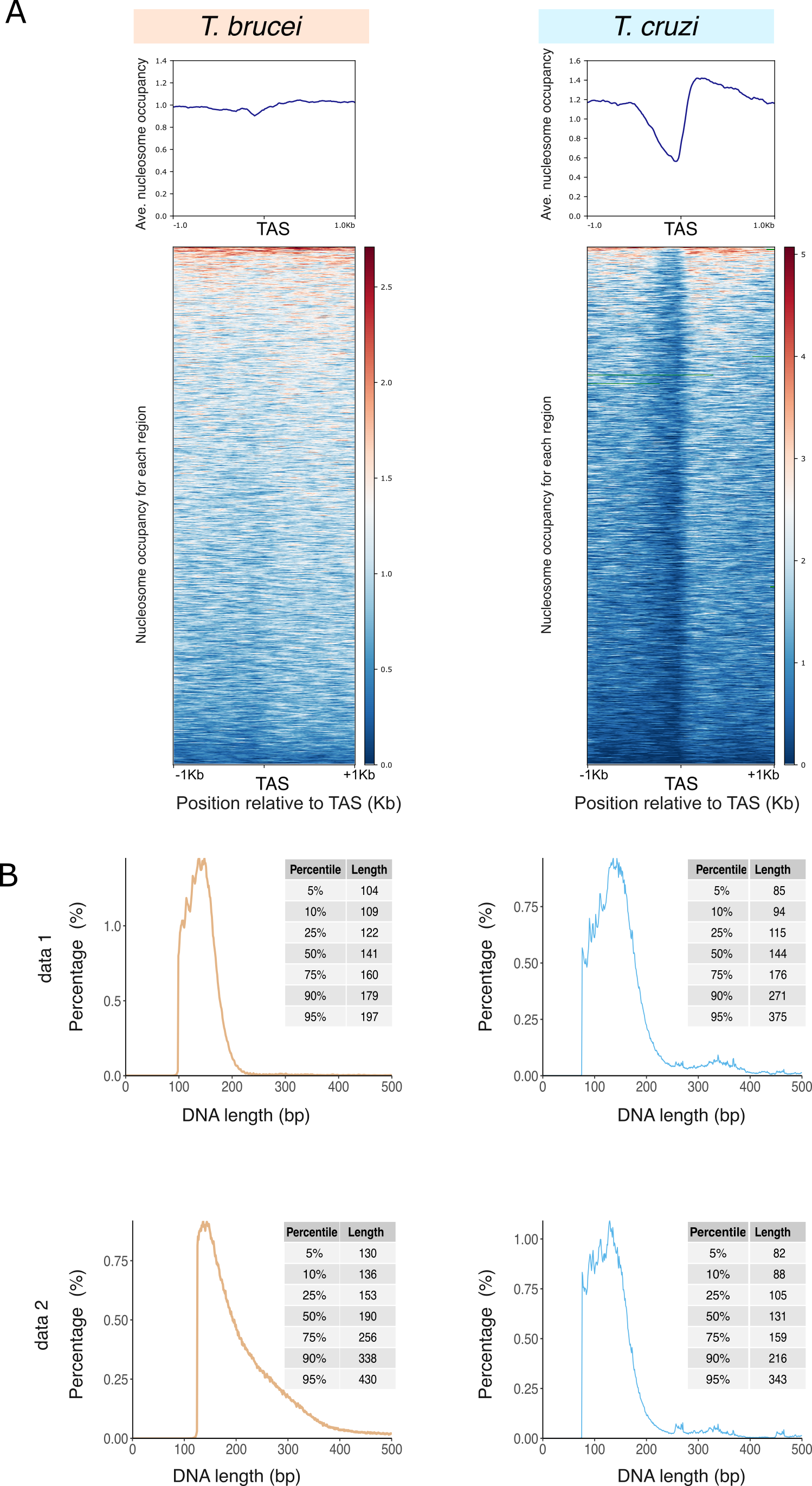

Supplement: S4 Fig — (A) Average H3 density (top panels) and heatmaps (bottom panels) for each region in a 1 kb window relative to the TAS. The signals scored for DNA molecules in the nucleosomal-size range (120–180 bp) are represented for T. brucei (GSM2586510) and T. cruzi (SRR14691957). (B) Length distribution histogram for sequenced DNA molecules for two replicate experiments of MNase-ChIP-seq for H3 for T. brucei 427, left panels: top (SRR13477532) and bottom (SRR13477532) and T. cruzi CL Brener, right panels: top (SRR14691958) and bottom (SRR14691957). (TIFF) [file pone.0343367.s004.tiff]

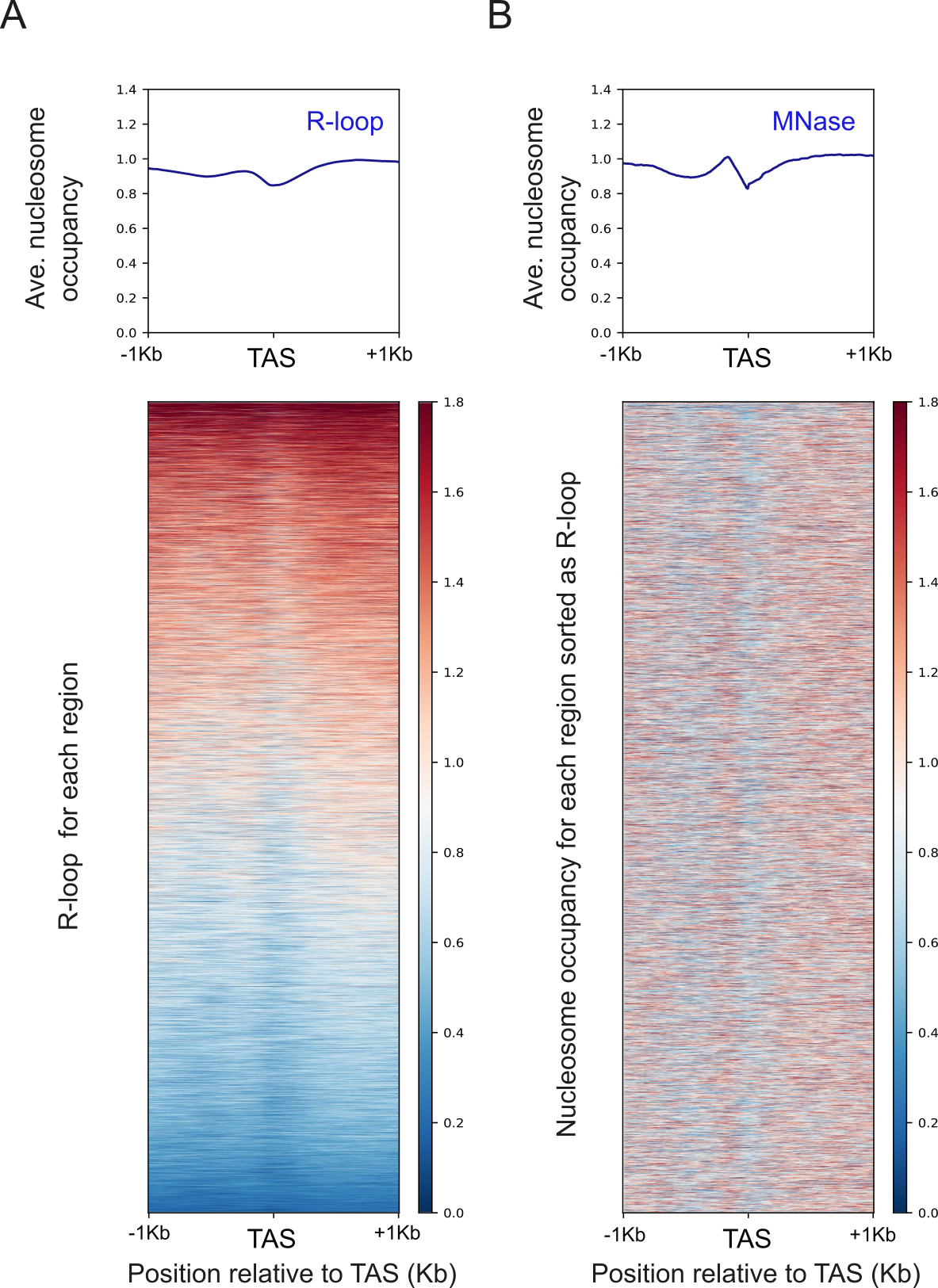

Supplement: S5 Fig — Average occupancy (top panels) and heatmaps (bottom panels) relative to TAS for (A) R-loops obtained from DRIP-seq (ERR12982995); and (B) Average nucleosome occupancy (GSM2179742). In both representations the regions plotted into heatmaps keep the same sorting, following the distribution of the R-loop signal, from higher (Red) to lower (Blue) density. (TIFF) [file pone.0343367.s005.tiff]

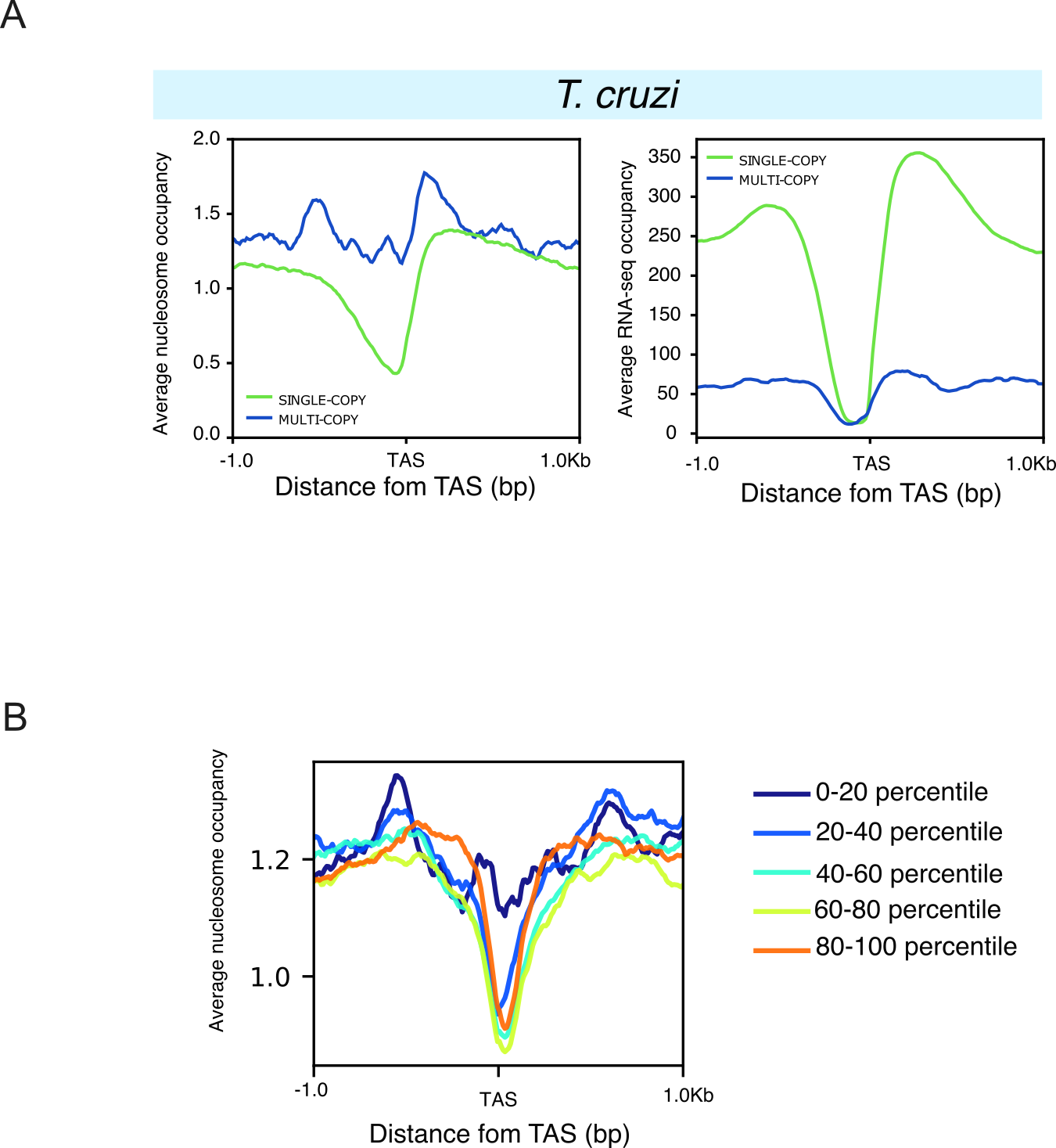

Supplement: S6 Fig — (A) Average histone H3 occupancy (left panel) and RNA-seq coverage (right panel) for T. cruzi CL Brener. (B) Average nucleosome occupancy of T. cruzi (GSM5363006) represented for the stratified quintiles according to RNA-seq expression using (SRX574894). (TIFF) [file pone.0343367.s006.tiff]
