# Supplementary material for: Beyond the transcript: Chromatin implications in trans-splicing in Trypanosomatids
Source: PLoS One. 2026 Feb 26;21(2):e0343367. doi: 10.1371/journal.pone.0343367 (PMC12944795; doi:10.1371/journal.pone.0343367)
Supplement: S1 Table — Complete information about original data sets and sample accession numbers. (DOCX) [file pone.0343367.s007.docx]

| **Table 1** | | | | | | |  | |
| --- | --- | --- | --- | --- | --- | --- | --- | --- |
| **MNase-seq data** | | | | | | |  | |
| **organism** | **Origin of data sets** | **technique** | **Project number** | **Sample accession number** | | **Length of sequenced reads** | **Figure in in which the data set is used** | |
| ***T. cruzi*** | Beati *et. al*, PLOS One, 2023 | MNase-seq | GSE176341 | GSM5363006 | | 50 | Fig 1 and S1 Fig (top panel) and S6B Fig | |
|  |  |  |  | GSM5363007 | | 50 | S1 Fig (bottom panel) and S2 Fig | |
| ***T. brucei*** | Maree *et. al*, Chromatin & Epigenetics, 2017 | MNase-seq | GSE90593 | GSM2407366 | | 50 | Fig 1, S1 Fig; and F4 | |
|  |  |  |  | GSM2407367 | | 50 | Fig S1 and S2 Fig | |
| ***L. major*** | Lombraña *et. al*, Cell Reports, 2016 | MNase-seq | GSE81991 | GSM2179742 | | 50 | Fig 1, S1 Fig (top panel) and S5 | |
|  |  |  |  | GSM2179741 | | 50 | S1 Fig (bottom panel) and S2 Fig | |
| **Histone H3 Inmunoprecipitation data** | | | | | | | | |
| ***T. brucei*** | Wedel *et. al,* EMBO Journal, 2017 | MNase-ChIP-seq | GSE98061 | GSM2586510 | | 99 | Fig 3 S4 Fig and S5 Fig | |
|  | Maree  *et. al*, NAR, 2022 | MNase-ChIP-seq (input) | GSE165034 | GSM5024927 | | 125 | Fig 2 and S3 Fig Low digestion | |
|  |  | MNase-ChIP-seq (input) |  | GSM5024915 | | 50-125 | Fig 2 and S3 Fig intermediate digestion | |
|  |  | MNase-ChIP-seq (input) |  | GSM5024921 | | 50-125 | Fig 2 and S3 Fig high digestion | |
|  |  | MNase-ChIP-seq |  | SRR13477532 | | 50-125 | S4A and B Figs, (bottom panel) and S5 Fig | |
| ***T. cruzi*** | Roson *et. al*, PLOS Pathogens, 2022 | MNase-ChIP-seq (IP) | PJNA733819 | SRR14691958 | | 76 | Fig 3, Fig 5 and S4 Fig (to panel) | |
|  |  | MNase-ChIP-seq (IP) |  | SRR14691957 | | 76 | S4A and B Fig, (bottom panel) and S6A Fig | |
| **Transcriptomic data used for UTR predictions** | | | | | | | | |
| ***T. cruzi*** | Li Y. *et al*, Pathogens, 2016 | RNA-seq | PRJNA251583 | SRX574894 |  | | S5A, right panel | |
|  |  |  |  | SRX574895 |  | |  | |
|  |  |  |  | SRX574896 |  | |  | |
| ***T. brucei*** | Muller *et al*, Nature, 2018 | RNA-seq | GSE100896 | GSM2695705 |  | |  | |
|  |  |  |  | GSM2695706 |  | |  | |
|  |  |  |  | GSM2695707 |  |  |  |  |
| ***L. major*** | Rastrojo *et al*, Scientific Reports, 2019 | RNA-seq | PRJEB27042 | ERR2604475 |  | |  | |
|  |  |  |  | ERR2604477 |  | |  | |
|  |  |  |  | ERR2604479 |  | |  | |
| **R-loop** | | | | | | | | |
| ***T. brucei*** | Briggs *et al*, NAR, 2018 | ﻿ DRIP-seq | PRJEB21868 | ERR2814820 |  | | Fig 4A | |
| ***L. major*** | Damasceno et al, *Nature communications,* 2025 | DRIP-seq | PRJEB75366 | ERR12982995 |  | | S5 Fig | |
| **RNAse pol II subunit** | | | | | | | | |
| **Rpb9** | Wedel *et. al,* EMBO Journal, 2017 | ChIP-seq | GSE98061 | SRR5466331 |  | | Fig 4C | |
|  | | | | | | | | |
| **FAIRE** | Lima  *et. al*, Epigenetics &chromatin, 2022 | FAIRE-seq | PRJNA763084 | SRR15902298 |  | | | Fig 5C (left) |
|  |  |  |  | SRR15902297 |  | | | Fig 5C (right) |
|  |  |  |  |  |  | | |  |
