# Supplementary material for: Beyond the transcript: Chromatin implications in trans-splicing in Trypanosomatids
Source: PLoS One. 2026 Feb 26;21(2):e0343367. doi: 10.1371/journal.pone.0343367 (PMC12944795; doi:10.1371/journal.pone.0343367)
Supplement: Attachment — Submitted filename: 159949_1_rebuttal_3547474_t6ljwx.docx [file pone.0343367.s010.docx]

**Manuscript number:** RC-2025-03124R

**Corresponding author(s):** Josefina Ocampo and Guillermo Alonso

## 1. General Statements [optional]

Our study provides the first comparative analysis of chromatin organization in trypanosomatids specially focusing on the trans-splicing acceptor sites (TASs). By applying the same bioinformatic pipeline we make a detailed comparison of their nucleosome landscapes. We also highlight the relevance of making appropriate comparisons between samples emphasizing the relevance of the MNase digestion achieved by them. Moreover, by correlating this information with publicly available data sets from different studies we make a comprehensive study of the potential implications of chromatin on gene expression and transcript maturation.

We are extremely grateful to the reviewers for helping us to improve our manuscript with their insightful and thorough revision. We tried to either address most of their suggestions or justify why we cannot fulfill their requests. We have also attended the suggestion provided upon submission to PLOS Pathogens. Particularly, we have made new UTR prediction for T. brucei using a more appropriate transcriptomic data of Lister 427 strain (detailed in methods section).

## 2. Description of the planned revisions

*Insert here a point-by-point reply that explains what revisions, additional experimentations and analyses are planned to address the points raised by the referees.*

* Please share the full analysis pipeline (ideally on GitHub/Zenodo) so the results are reproducible from raw reads to plots.

-Most of the pipelines used in this manuscript are already published (Beati PlosONE, 2023 and Radio S, 2018) and publicly available at GiHub (https://github.com/paulati/nucleosome). However, we take the reviewer’s suggestion, and we are updating every detailed used here in a source code that we will make available before the completion of this revision process.

* As an optional but helpful expansion, consider including additional datasets (other life stages, BSF MNase-seq, ATAC-seq, DRIP-seq) where available to strengthen comparative claims.

We are now including a new figure 4 and a supplemental figure 5 including DRIP-seq and Rpb9 ChIP-seq for *T. brucei* (revised Fig 4) and DRIP-seq for *L. major* (S5 Fig). Additionally, we added FAIRE-seq data to previous Fig 4 now Fig 5 (revised Fig 5C).

We are analyzing ATAC-seq data for *T. brucei*.

Regarding BSF MNase-seq, the original article by Mareé 2017 claims that there is not significant difference for average chromatin organization between the two life forms; therefore, is not worth including that analysis.

Following the reviewer’s recommendation, we will provide a more detail description in the final revision comparing how our pipelines represent an improvement relative to the original articles.

## 3. Description of the revisions that have already been incorporated in the transferred manuscript

*Please insert a point-by-point reply describing the revisions that were already carried out and included in the transferred manuscript. If no revisions have been carried out yet, please leave this section empty.*

See Below, a point-by-point response to the reviewers:

**Reviewer #1 (Evidence, reproducibility and clarity (Required)):** 

This study explores chromatin organization around trans-splicing acceptor sites (TASs) in the trypanosomatid parasites Trypanosoma cruzi, T. brucei and Leishmania major. By systematically re-analyzing MNase-seq and MNase-ChIP-seq datasets, the authors conclude that TASs are protected by an MNase-sensitive complex that is, at least in part, histone-based, and that single-copy and multi-copy genes display differential chromatin accessibility. Altogether, the data suggest a common chromatin landscape at TASs and imply that chromatin may modulate transcript maturation, adding a new regulatory layer to an unusual gene-expression system.

I value integrative studies of this kind and appreciate the careful, consistent data analysis the authors implemented to extract novel insights. That said, several aspects require clarification or revision before the conclusions can be robustly supported. My main concerns are listed below, organized by topic/result section. 

**Results - "There is a distinctive average nucleosome arrangement at the TASs in TriTryps":**
* You state that "In the case of L. major the samples are less digested." However, Supplementary Fig. S1 suggests that replicate 1 of L. major is less digested than the T. brucei samples, while replicate 2 of L. major looks similarly digested. Please clarify which replicates you reference and correct the statement if needed.

The reviewer has a good point. We made our statement based on the value of the maximum peak of the sequenced DNA molecules, which in general is a good indicative of the extension of the digestion achieved by the sample (Cole H, NAR, 2011).

As the reviewer correctly points, we should have also considered the length of the DNA molecules in each percentile. However, in this case both, *T. brucei’s and L major’s* samples were gel purified before sequencing, and it is hard to know exactly what fragments were left behind in each case. Therefore, it is better to avoid over concluding on that regard.

We have now comment on this in the main manuscript, and we have clarified which data set we used in each case in the figure legends and in Table S1.

* It appears you plot one replicate in Fig. 1b and the other in Suppl. Fig. S2. Please indicate explicitly which replicate is in each plot. For T. brucei, the NDR upstream of the TAS is clearer in Suppl. Fig. S2 while the TAS protection is less prominent; based on your digestion argument, this should correspond to the more-digested replicate. Please confirm.

The replicates used for the construction of each figure were explicitly indicated in Table S1. Although we have detailed in the table the original publication, the project and accession number for each data set, the reviewer is correct that in this case it was still not completely clear to which length distribution heatmap each sample was associated with. To avoid this confusion, we have now added the accession number for each data set to the figure legends and clarified in Table S1. Regarding the reviewer’s comment on the correspondence between the observed TAS protection and the extent of samples digestion, he/she is correct that for a more digested sample we would expect a clearer NDR. In this case, the difference in the extent of digestion between these two samples is minor, as observed the length of the main peak in the length distribution histogram for sequenced DNA molecules is the same. These two samples GSM5363006, represented in Fig1 B, and GSM5363007, represented in S2 Fig, belong to the same original paper (Maree et al 2017), and both were gel-purified before sequencing. Therefore, any difference between them could not only be the result of a minor difference in the digestion level achieved in each experiment, but could be also biased by the fragments included or not during gel purification. Therefore, I would not over conclude about TAS protection from this comparison. We have now included a brief comment on this, in the figure discussion.

* The protected region around the TAS appears centered on the TAS in T. brucei but upstream in L. major. This is an interesting difference. If it is technical (different digestion or TAS prediction offset), explain why; if likely biological, discuss possible mechanisms and implications.

We appreciate the reviewer suggestion. We cannot assure if it is due to technical or biological reasons, but there is evidence that *L. major ‘s* genome has a different dinucleotide content, and it might have an impact on nucleosome assembly. We have now added a comment about this observation in the final discussion of the manuscript. Additionally, we analyzed DRIP-seq data for *L. major,* recently published doi: 10.1038/s41467-025-56785-y, and we observed that the R-loop footprint co-localized with the MNase-protected region upstream of the TAS (new S5 Fig), suggesting that the shift is not related to the MNase-seq technique.

**Results - "An MNase sensitive complex occupies the TASs in T. brucei":** 
* The definition of "MNase activity" and the ordering of samples into Low/Intermediate/High digestion are unclear. Did you infer digestion levels from fragment distributions rather than from controlled experimental timepoints? In Suppl. Fig. S3a it is not obvious how "Low digestion" was defined; that sample's fragment distribution appears intermediate. Please provide objective metrics (e.g., median fragment length, fraction 120-180 bp) used to classify digestion levels.

As the reviewer suggests, the ideal experiment would be to perform a time course of MNase reaction with all the samples in parallel, or to work with a fix time point adding increasing amounts of MNase. However, even when making controlled experimental timepoints, you need to check the length distribution histogram of sequenced DNA molecules to be sure which level of digestion you have achieved.

In this particular case, we used publicly available data sets to make this analysis. We made an arbitrary definition of low, intermediate and high level of digestion, not as an absolute level of digestion, but as a comparative output among the tested samples. We based our definition on the comparison of **the main peak in length distribution heatmaps because this parameter is the best metric to estimate the level of digestion of a given sample. This main peak represents the percentage of the total DNA sequenced that contains the predominant length in the sample tested.** Hence, we considered:

**low digestion**: when the main peak is longer than the expected protection for a nucleosome (longer than 150 bp). We expect this sample to contain additional longer bands that correspond to less digested material.

**intermediate digestion**, when the main peak is the expected for the nucleosome core-protection (˜146-150bp).

**high digestion**, when the main peak is shorter than that (shorter than 146 bp). This case is normally accompanied by a bigger dispersion in fragment sizes.

To do this analysis, we chose samples that render different MNase protection of the TAS when plotting all the sequenced DNA molecules relative to this point and we used this protection as a predictor of the extent of sample digestion (Figure 2). To corroborate our hypothesis, that the degree of TAS protection was indeed related to the extent of the MNase digestion of a given sample, we looked at the length distribution histogram of the sequenced DNA molecules in each case. It is the best measurement of the extent of the digestion achieved, especially, when sequencing the whole sample without any gel purification and representing all the reads in the analysis as we did. The only caveat is with the sample called “intermediate digestion 1” that belongs to the original work of Mareé 2017, since only this data set was gel purified. To avoid this problem, we decided to remove this data from figures 2 and S3. In summary, the 3 remaining samples comes from the same lab, and belong to the same publication (Mareé 2022). These sample are the inputs of native MNase ChIp-seq, obtain the same way, totally comparable among each other.

* Several fragment distributions show a sharp cutoff at ~100-125 bp. Was this due to gel purification or bioinformatic filtering? State this clearly in Methods. If gel purification occurred, that can explain why some datasets preserve the MNase-sensitive region.

The sharp cutoff is neither due to gel purification nor bioinformatic filtering, it is just due to the length of the paired-end read used in each case. In earlier works the most common was to sequence only 50bp, with the improvement of technologies it went up to 75,100 or 125 bp. We have now clarified in Table S1 the length of the paired-reads used in each case when possible.

* Please reconcile cases where samples labeled as more-digested contain a larger proportion of >200 bp fragments than supposedly less-digested samples; this ordering affects the inference that digestion level determines the loss/preservation of TAS protection. Based on the distributions I see, "Intermediate digestion 1" appears most consistent with an expected MNase curve - please confirm and correct the manuscript accordingly.

As explained above, it's a common observation in MNase digestion of chromatin that more extensive digestion can still result in a broad range of fragment sizes, including some longer fragments. This seemingly counterintuitive result is primarily due to the non-uniform accessibility of chromatin and the sequence preference of the MNase enzyme, which has a preference for AT reach sequences.

The rationale of this is as follows: when you digest chromatin with MNase and the objective is to map nucleosomes genome-wide, the ideal situation would be to get the whole material contained in the mononucleosome band. Given that MNase is less efficient to digest protected DNA but, if the reaction proceeds further, it always ends up destroying part of it, the result is always far from perfect. The better situation we can get, is to obtain samples were ˜80% of the material is contained in the mononucloesome band. **And here comes the main point:** even in the best scenario, you always get some additional longer bands, such as those for di or tri nucleosomes. If you keep digesting, you will get less than 80 % in the nucleosome band and, those remaining DNA fragments that use to contain di and tri nucleosomes start getting digested as well, originating a bigger dispersion in fragments sizes. How do we explain persistence of Long Fragments? The longest fragments (di-, tri-nucleosomes) that persist in a highly digested sample are the ones that were originally most highly protected by proteins or higher-order structure, or by containing a poor AT sequence content, making their linker DNA extremely resistant to initial cleavage. Once the majority of the genome is fragmented, these few resistant longer fragments become a more visible component of the remaining population, contributing to a broader size dispersion. Hence, you end up observing a bigger dispersion in length distributions in the final material. Bottom line, it is not a good practice to work with under or overdirected samples. Our main point is to emphasize that especially when comparing samples, it important to compare those with comparable levels of digestion. Otherwise, a different sampling of the genome will be represented in the remaining sequenced DNA.

**Results - "The MNase sensitive complexes protecting the TASs in T. brucei and T. cruzi are at least partly composed of histones":**
* The evidence that histones are part of the MNase-sensitive complex relies on H3 MNase-ChIP signal in subnucleosomal fragment bins. This seems to conflict with the observation (Fig. 1) that fragments protecting TASs are often nucleosome-sized. Please reconcile these points: are H3 signals confined to subnucleosomal fragments flanking the TAS while the TAS itself is depleted of H3? Provide plots that compare MNase-seq and H3 ChIP signals stratified by consistent fragment-size bins to clarify this.

What we learned from other eukaryotic organisms that were deeply studied, such as yeast, is that NDRs are normally generated at regulatory points in the genome. In this sense, yeast tRNA genes have a complex with a bootprint smaller than a nucleosome formed by TFIIIC-TFIIB (Nagarajavel, doi: 10.1093/nar/gkt611). On the other hand, many promotor regions have an MNase-sensitive complex with a nucleosome-size footprint, but it does not contain histones (Chereji, et al 2017, doi:10.1016/j.molcel.2016.12.009). The reviewer is right that from Figure 1 and S2 we could observe that the footprint of whatever occupies the TAS region, especially in *T. brucei*, is nucleosome-size. However, it only shows the size, but it doesn’t prove the nature of its components. Nevertheless, those are only MNase-seq data sets. Since it does not include a precipitation with specific antibodies, we cannot confirm the protecting complex is made up by histones. In parallel, a complementary study by Wedel 2017, from Siegel’s lab, shows that using a properly digested sample and further immunoprecipitating with a-H3 antibody, the TAS is not protected by nucleosomes at least not when analyzing nucleosome size-DNA molecules. Besides, Briggs et. al 2018 (doi: 10.1093/nar/gky928) showed that at least at intergenic regions H3 occupancy goes down while R-loops accumulation increases. We have now added a new figure 4 replotting R-loops and MNase-ChIP-seq for H3 relative to our predicted TAS showing this anti-correlation and how it partly correlates with MNase protection as well. As a control we show that Rpb9 trends resembles H3 as Siegel’s lab have shown in Wedel 2018. Moreover, we analyzed redate from a recently published paper (doi: 10.1038/s41467-025-56785-y) added a new supplemental figure 5 showing that a similar correlation between MNase protection and R-loop footprint occurs in *L. major* (S5 Fig).

* Please indicate which datasets are used for each panel in Suppl. Fig. S4 (e.g., Wedel et al., Maree et al.), and avoid calling data from different labs "replicates" unless they are true replicates.

In most of our analysis we used real replicated experiments. Such is the case MNase-seq data used in Figure 1, with the corresponding replicate experiments used in Figure S2; *T. cruzi* MNase-ChIP-seq data used in Figure 3B and new 5A with the respective replicate used in Figures S4 and now S6 in the revised manuscript. The only case in which we used experiments coming from two different laboratories, is in the case of MNase-ChIP-seq for H3 from *T. brucei.* Unfortunately, there are only two public data sets coming each of them from different laboratories. The samples used in Fig 3 (from Siegel’s lab) whether the IP from H3 represented in S4 and S5 (S6 in the updated version) comes from another lab (Patterton’s). To be more rigorous, we now call them data 1 and 2 when comparing this particular case.

The reviewer is right that in this particular case one is native chromatin (Pattertons’) while the other one is crosslinked (Siegel’s). We have now clarified it in the main text that unfortunately we do not count on a replicate but even under both condition the result remains the same, and this is compatible with my own experience, were crosslinking does not affect the global nucleosome patterns (compared nucleosome organization from crosslinked chromatin MNase-seq inputs Chereji, Mol Cell, 2017 doi: 10.1016/j.molcel.2016.12.009 and native MNase-seq from Ocampo, NAR, 2016 doi: 10.1093/nar/gkw068).

* Several datasets show a sharp lower bound on fragment size in the subnucleosomal range (e.g., ~80-100 bp). Is this a filtering artifact or a gel-size selection? Clarify in Methods and, if this is an artifact, consider replotting after removing the cutoff.

We have only filtered adapter dimmer or overrepresented sequences when needed. In Figures 2 and S3 we represented all the sequenced reads. In other figures when we sort fragments sizes *in silico,* such as nucleosome range, dinucleosome or subnucleosome size, we make a note in the figure legends. What the reviewer points is related to the length of the sequence DNA fragment in each experiment. As we explained above, the older datasets were performed with 50 bp paired-end reads, the newer ones are 75, 100 or 125bp. This is information is now clarified in Table S1.

**Results - "The TASs of single and multi-copy genes are differentially protected by nucleosomes":**

* Discuss how low or absent expression of multigene families affects TAS annotation (which relies on RNA-seq) and whether annotation inaccuracies could bias the observed chromatin differences.

The mapping of occurrence and annotations that belong to repetitive regions has great complexity. UTRme is specially designed to avoid overcalling those sites. In other words, there is a chance that we could be underestimating the number of predicted TASs at multicopy genes. Regarding the impact on chromatin analysis, we cannot rule out that it might have an impact, but the observation favors our conclusion, since even when some TASs at multicopy genes can remain elusive, we observe more nucleosome density at those places.

* The statement that multi-copy genes show an "oscillation" between AT and GC dinucleotides is not clearly supported: the multi-copy average appears noisier and is based on fewer loci. Please tone down this claim or provide statistical support that the pattern is periodic rather than noisy. We have fixed this now in the preliminary revised version

* How were multi-copy genes defined in T. brucei? Include the classification method in Methods. This classification was done the same way it was explained for *T. cruzi.* However, decided to remove the supplemental figure that included this sorting.

**Genomes and annotations:** 
* If transcriptomic data for the Y strain was used for T. cruzi, please explain why a Y strain genome was not used (e.g., Wang et al. 2021 GCA_015033655.1), or justify the choice. For T. brucei, consider the more recent Lister 427 assembly (Tb427_2018) from TriTrypDB. Use strain-matched genomes and transcriptomes when possible, or discuss limitations.

The most appropriate way to analyze high throughput data, is to align it to the same genome where the experiments were conducted. This was clearly illustrated in a previous publication from our group where we explained how data from the hybrid CL Brener strain should be analyzed. In the past, a common practice was to use only Esmeraldo-like genome for simplicity, but this resulted in output artifacts. Therefore, we aligned it to CL Brener genome and then focused the main analysis on the Esmeraldo haplotype (Beati Plos ONE, 2023). Ideally, we should have counted on transcriptomic data for the same strain (CL Brener or Esmeraldo). Since this was not the case at that moment, we used data from Y strain that belongs to the same DTU with Esmeraldo.

In the case of *T. brucei*, when we started our analysis and the software code for UTRme was written, the previous version of the genome was available. Upon 2018 version came up, we checked chromatin parameters and observed that the main findings did not change. Therefore, we continue working with our previous setups.

**Reproducibility and broader integration**: 
* Please share the full analysis pipeline (ideally on GitHub/Zenodo) so the results are reproducible from raw reads to plots.

We are preparing a full pipeline in GitHub. We will make it available before manuscript full revision

* As an optional but helpful expansion, consider including additional datasets (other life stages, BSF MNase-seq, ATAC-seq, DRIP-seq) where available to strengthen comparative claims.

We are now including a new figure 4 and a supplemental figure 5 including DRIP-seq and Rp9 ChIP-seq for *T. brucei* (revised Fig 4) and DRIP-seq for *L. major* (S5 Fig). Additionally, we added FAIRE-seq data to previous Fig 4 now Fig 5 (revised Fig 5C).

We are analyzing ATAC-seq data for *T. brucei*.

Regarding BSF MNase-seq, the original article by Mareé 2017 claims that there is not significant difference for average chromatin organization between the two life forms; therefore, is not worth including that analysis.

**Optional analyses that would strengthen the study**: 
* Stratify single-copy genes by expression (high / medium / low) and examine average nucleosome occupancy at TASs for each group; a correlation between expression and NDR depth would strengthen the functional link to maturation.

We have now included a panel in supplemental figure 5 (now revised S6), showing the concordance for chromatin organization of stratified genes by RNA-seq levels relative to TAS.

**Minor / editorial comments:** 
* In the Introduction, the sentence "transcription is initiated from dispersed promoters and in general they coincide with divergent strand switch regions" should be qualified: such initiation sites also include single transcription start regions. We have clarified this in the preliminary revised version

* Define the dotted line in length distribution plots (if it is not the median, please clarify) and consider placing it at 147 bp across plots to ease comparison. The dotted line is just to indicate where the maximum peak is located. It is now clarified in figure legends.

* In Suppl. Fig. 4b "Replicate2" the x-axis ticks are misaligned with labels - please fix. We have now fixed the figure. Thanks for noticing this mistake.

* Typo in the Introduction: "remodellingremodeling" → "remodeling
Thanks for noticing this mistake, it is fixed in the current version of the manuscript

**Referee cross-commenting** 
Comment 1: I think Reviewer #2 and Reviewer #3 missed that they authors of this manuscript do cite and consider the results from Wedel at al. 2017. They even re-analysed their data (e.g. Figure 3a).
I second Reviewer #2 comment indicating that the inclusion of a schematic figure to help readers visualize and better understand the findings would be an important addition.

Comment 2: I agree with Reviewer #3 that the use of different MNase digestion procedures in the different datasets have to be considered. On the other hand, I don't think there is a problem with figure 1 showing an MNase-protected TAS for T. brucei as it is based on MNase-seq data and reproduces the reported results (Maree et al. 2017). What the Siegel lab did in Wedel et al. 2017 was MNase-ChIPseq of H3 showing nucleosome depletion at TAS, but both results are not necessary contradictory: There could still be something else (which does not contain H3) sitting on the TAS protecting it from MNase digestion.

Reviewer #1 (Significance (Required)): 

This study provides a systematic comparative analysis of chromatin landscapes at trans-splicing acceptor sites (TASs) in trypanosomatids, an area that has been relatively underexplored. By re-analyzing and harmonizing existing MNase-seq and MNase-ChIP-seq datasets, the authors highlight conserved and divergent features of nucleosome occupancy around TASs and propose that chromatin contributes to the fidelity of transcript maturation.
The significance lies in three aspects: 
1. Conceptual advance: It broadens our understanding of gene regulation in organisms where transcription initiation is unusual and largely constitutive, suggesting that chromatin can still modulate post-transcriptional processes such as trans-splicing.
2. Integrative perspective: Bringing together data from T. cruzi, T. brucei and L. major provides a comparative framework that may inspire further mechanistic studies across kinetoplastids.
3. Hypothesis generation: The findings open testable avenues about the role of chromatin in coordinating transcript maturation, the contribution of DNA sequence composition, and potential interactions with R-loops or RNA-binding proteins.
Researchers in parasitology, chromatin biology, and RNA processing will find it a useful resource and a stimulus for targeted experimental follow-up.

My expertise is in gene regulation in eukaryotic parasites, with a focus on bioinformatic analysis of high-throughput sequencing data

**Reviewer #2 (Evidence, reproducibility and clarity (Required)):** 

Siri et al. perform a comparative analysis using publicly available MNase-seq data from three trypanosomatids (T. brucei, T. cruzi, and Leishmania), showing that a similar chromatin profile is observed at TAS (trans-splicing acceptor site) regions. The original studies had already demonstrated that the nucleosome profile at TAS differs from the rest of the genome; however, this work fills an important gap in the literature by providing the most reliable cross-species comparison of nucleosome profiles among the tritryps. To achieve this, the authors applied the same computational analysis pipeline and carefully evaluated MNase digestion levels, which are known to influence nucleosome profiling outcomes.

In my view, the main conclusion is that the profiles are indeed similar-even when comparing T. brucei and T. cruzi. This was not clear in previous studies (and even appeared contradictory, reporting nucleosome depletion versus enrichment) largely due to differences in chromatin digestion across these organisms. The manuscript could be improved with some clarifications and adjustments:

1. The authors state from the beginning that available MNase data indicate altered nucleosome occupancy around the TAS. However, they could also emphasize that the conclusions across the different trypanosomatids are inconsistent and even contradictory: NDR in T. cruzi versus protection-in different locations-in T. brucei and Leishmania.

We start our manuscript by referring to the first MNase-seq data sets publicly available for each TriTryp and we point that one of the main observations, in each of them, is the occurrence of a change in nucleosome density or occupancy at intergenic regions. In *T. cruzi*, in a previous publication from our group, we stablished that this intergenic drop in nucleosome density occurs near the trans-splicing acceptor site. In this work, we extend our study to the other members of TriTryps: *T. brucei* and *L. major*.

In *T. brucei* the papers from Patterton’s lab and Siegel’s lab came out almost simultaneously in 2017. Hence, they do not comment on each other’s work. The first one claims the presence of a well-positioned nucleosome at the TAS by using MNase-seq, while the second one, shows an NDR at the TAS by using MNase-ChIP-seq. However, we do not think they are contradictory, or they have inconsistency. We brought them together along the manuscript because we think these works can provide complementary information.

On one hand, we infer data from Pattertons lab is slightly less digested than the sample from Siegel’s lab. Therefore, we discuss that this moderate digestion must be the reason why they managed to detect an MNase protecting complex sitting at the TAS (Figure 1). On the other hand, Sigel’s lab includes an additional step by performing MNase-ChIP-seq, showing that when analyzing nucleosome size fragments, histones are not detected at the TAS. Here, we go further in this analysis on figure 3, showing that only when looking at subnucleosome-size fragments, we can detect histone H3. And this is also true for *T. cruzi.*

By integrating every analysis in this work and the previous ones, we propose that TASs are protected by an MNase-sensitive complex (proved in Figure 2). This complex most likely is only partly formed by histones, since only when analyzing sub-nucleosomes size DNA molecules we can detect histone H3 (Figure 3). To be sure that the complex is not entirely made up by histones, future studies should perform an MNse-ChIP-seq with less digested samples. However, it was previously shown that R-loops are enriched at those intergenic NDRs (Briggs, 2018 doi: 10.1093/nar/gky928) and that R-loops have plenty of interacting proteins (Girasol, 2023 10.1093/nar/gkad836). Therefore, most likely, this MNase-sensitive complexed have a hybrid nature made up by H3 and some other regulatory molecules, possibly involved in trans-splicing. We have now added a new figure 4 showing R-loop co-localization with the NDR.

Regarding the comparison between different organisms, after explaining the sensitivity to MNase of the TAS protecting complex, we discuss that when comparing equally digested samples *T. cruzi* and *T. brucei* display a similar chromatin landscape with a mild NDR at the TAS (See *T. cruzi* represented in Figure 1 compared to *T. brucei* represented in Intermediate digestion 2 in Figure 2, intermediate digestion in the revised manuscript). Unfortunately, we cannot make a good comparison with *L. major*, since we do not count on a similar level of digestion. However, by analyzing a recently published DRIP-seq data-set for *L. major* we show that R-loop signal co localize with MNase-protection in a similar way (new S5 Fig).

2. Another point that requires clarification concerns what the authors mean in the introduction and discussion when they write that trypanosomes have "...poorly organized chromatin with nucleosomes that are not strikingly positioned or phased." On the other hand, they also cite evidence of organization: "...well-positioned nucleosome at the spliced-out region.. in Leishmania (ref 34)"; "...a well-positioned nucleosome at the TASs for internal genes (ref37)"; "...a nucleosome depletion was observed upstream of every gene (ref 35)." Aren't these examples of organized chromatin with at least a few phased nucleosomes? In addition, in ref 37, figure 4 shows at least two (possibly three to four) nucleosomes that appear phased. In my opinion, the authors should first define more precisely what they mean by "poorly organized chromatin" and clarify that this interpretation does not contradict the findings highlighted in the cited literature.

For a better understanding of nucleosome positioning and phasing I recommend the review: Clark 2010 doi:10.1080/073911010010524945, Figure 4. Briefly, in a cell population there are different alternative positions that a given nucleosome can adopt. However, some are more favorable. When talking about favorable positions, we refer to the coordinates in the genome that are most likely covered by a nucleosome and are predominant in the cell population. Additionally, nucleosomes could be phased or not. This refers not only the position in the genome, but to the distance relative to a given point. In yeast, or in highly transcribed genes of more complex eukaryotes, nucleosomes are regularly spaced and phased relative to the transcription start site (TSS) or to the +1 nucleosome (Ocampo, NAR, 2016, doi:10.1093/nar/gkw068). In trypanosomes, nucleosomes have some regular distribution when making a browser inspection but, given that they are not properly phased with respect to any point, it is almost impossible to make a spacing estimation from paired-end data. This is also consistent with a chromatin that is transcribed in an almost constitutive manner.

As the reviewer mention, we do site evidence of organization. We think the original observations are correct, but we do not fully agree with some of the original statements. In this manuscript our aim is to take the best we learned from their original works and to make a constructive contribution adding to the original discussions. In this regard, in trypanosomes there are some conserved patterns in the chromatin landscape, but their nucleosomes are far from being well-positioned or phased. For a better understanding, compare the variations observed in the y axis when representing av. nucleosome occupancy in yeast with those observed in trypanosomes and you will see that the troughs and peaks are much more prominent in yeast than the ones observed in any TryTryp member.

Following the reviewer’s suggestion we have now clarified this in the main text

3. The paper would also benefit from the inclusion of a schematic figure to help readers visualize and better understand the findings. What is the biological impact of having nucleosomes, di-nucleosomes, or sub-nucleosomes at TAS? This is not obvious to readers outside the chromatin field. For example, the following statement is not intuitive: "We observed that, when analyzing nucleosome-size (120-180 bp) DNA molecules or longer fragments (180-300 bp), the TASs of either T. cruzi or T. brucei are mostly nucleosome-depleted. However, when representing fragments smaller than a nucleosome-size (50-120 bp) some histone protection is unmasked (Fig. 3 and Fig. S4). This observation suggests that the MNase sensitive complex sitting at the TASs is at least partly composed of histones." Please clarify.

We appreciate the reviewer’s suggestion to make a schematic figure. We have now added a new Figure 6.

Regarding the biological impact of having mono, di or subnucleosome fragments, it is important to unveil the fragment size of the protected DNA to infer the nature of the protecting complex. In the case of tRNA genes in yeast, at pol III promoters they found footprints smaller than a nucleosome size that ended up being TFIIB-TFIIC (Nagarajavel, doi: 10.1093/nar/gkt611). Therefore, detecting something smaller than a nucleosome might suggest the binding of trans-acting factors different than histones or involving histones in a mixed complex. These mixed complexes are also observed, and that is the case of the centromeric nucleosome which has a very peculiar composition (Ocampo and Clark, Cells Reports, 2015). On the other hand, if instead we detect bigger fragments, it could be indicative of the presence of bigger protecting molecules or that those regions are part of higher order chromatin organization still inaccessible for MNase linker digestions.

Here we show on 2Dplots, that complex or components protecting the TAS have nucleosome size, but we cannot assure they are entirely made up by histones, since, only when looking at subnucleosome-size fragments, we are able to detect histone H3. We have now added part of this explanation to the discussion.

By integrating every analysis in this work and the previous ones, we propose that the TAS is protected by an MNase-sensitive complex (Figure 2). This complex most likely is only partly formed by histones, since only when analyzing sub-nucleosomes size DNA molecules we can detect histone H3 (Figure 3). As explained above, to be sure that the complex is not entirely made up by histones, future studies should perform an MNse-ChIP-seq with less digested samples. However, it was previously shown that R-loops are enriched at those intergenic NDRs (Briggs 2018) and that R-loops have plenty of interacting proteins (Girasol, 2023). Therefore, most likely, this MNase-sensitive complexed have a hybrid nature made up by H3 and some other regulatory molecules. We have now added a new figure 4 showing R-loop partial co-localization with MNase protection.

Some references are missing or incorrect: we will make a thorough revision

"In trypanosomes, there are no canonical promoter regions." - please check Cordon-Obras et al. (Navarro's group). Thank you for the appropriate suggestion. We have now added this reference

Please, cite the study by Wedel et al. (Siegel's group), which also performed MNase-seq analysis in T. brucei.

We understand that reviewer number 2# missed that we cited this reference and that we did used the raw data from the manuscript of Wedel et. al 2017 form Siegel’s group. We used the MNase-ChIP-seq data set of histone H3 in our analysis for Figures 3, S4 and S6 (in the revised version), also detailed in table S1. To be even more explicit, we have now included the accession number of each data set in the figure legends.

Figure-specific comments: 

Fig. S3: Why does the number of larger fragments increase with greater MNase digestion? Shouldn't the opposite be expected?

This a good observation. As we also explained to reviewer#1:

It's a common observation in MNase digestion of chromatin that more extensive digestion can still result in a broad range of fragment sizes, including some longer fragments. This seemingly counter-intuitive result is primarily due to the non-uniform accessibility of chromatin and the sequence preference of the MNase enzyme.

The rationale of this is as follows: when you digest chromatin with MNase and the objective is to map nucleosomes genome-wide, the ideal situation would get the whole material contained in the mononucleosome band. Given that MNase is less efficient to digest protected DNA but, if the reaction proceeds further, it always ends up destroying part of it, the result is always far from perfect. The better situation we can get, is to obtain samples were ˜80% of the material is contained in the mononucloesome band. **And here comes the main point:** even in the best scenario, you always have some additional longer bands, such as those for di or tri nucleosomes. If you keep digesting, you will get less than 80 % in the nucleosome band and, those remaining DNA fragments that use to contain di and tri nucleosomes start getting digested as well originating a bigger dispersion in fragments sizes. How do we explain persistence of Long Fragments? The longest fragments (di-, tri-nucleosomes) that persist in a highly digested sample are the ones that were originally most highly protected by proteins or higher-order structure, making their linker DNA extremely resistant to initial cleavage. Once most of the genome is fragmented, these few resistant longer fragments become a more visible component of the remaining population, contributing to a broader size dispersion. Hence, there you end up having a bigger dispersion in length distributions in the final material. Bottom line, it is not a good practice to work with under or overdirected samples. Our main point is to emphasize that especially when comparing samples, it important to compare those with comparable levels of digestion. Otherwise, a different sampling of the genome will be represented in the remaining sequenced DNA
Fig. S5B: Why not use MNase conditions under which T. cruzi and T. brucei display comparable profiles at TAS? This would facilitate interpretation.

The reviewer made a reasonable observation. The reason why we used MNase-ChIP-seq instead of just MNase to test occupancy at TAS at the subsets of genes, is because we intended to be more certain if we were talking about the presence of histones or something else. By using IP for histone H3 we can see that at multi-copy genes this protein is present when looking at nucleosome-size fragments. Additionally, as shown in figure S4b, length distribution histograms are also similar for the compared IPs.

Minor points: 

There are several typos throughout the manuscript.

Thanks for the observation. We will check carefully.
Methods: "Dinucelotide frecuency calculation."  We will add a code in the upcoming GitHub

**Reviewer #3 (Evidence, reproducibility and clarity (Required)):** 

The authors analyzed publicly accessible MNase-seq data in TriTryps parasites, focusing on the chromatin structure around trans-splicing acceptor sites (TASs), which are vital for processing gene transcripts. They describe a mild nucleosome depletion at the TAS of T. cruzi and L. major, whereas a histone-containing complex protects the TASs of T. brucei. In the subsequent analysis of T. brucei, they suggest that a Mnase-sensitive complex is localised at the TASs. For single-copy versus multi-copy genes, the authors show different di-nucleotide patterns and chromatin structures. Accordingly, they propose this difference could be a novel mechanism to ensure the accuracy of trans-splicing in these parasites.

Before providing an in- depth review of the manuscript, I note that some missing information would have helped in assessing the study more thoroughly; however, in the light of the available information, I provide the following comments for consideration.

The numbering of the figures, including the figure legends, is missing in the PDF file. This is essential for assessing the provided information.

We apologized for not including the figure numbers in the main text, although they are in the right place when called in the text. The omission was unwillingly made when figure legends were moved to the bottom of the main text. This is now fixed in the updated version of the manuscript.

The publicly available Mnase- seq data are manyfold, with multiple datasets available for T. cruzi, for example. It is unclear from the manuscript which dataset was used for which figure. This must be clarified.

This was detailed in Tabe S1. We have now replaced the table by an improved version, and we have also included the accession number of each data set used in the figure legends.

Why do the authors start in figure 1 with the description of an MNase- protected TAS for T.brucei, given that it has been clearly shown by the Siegel lab that there is a nucleosome depletion similar to other parasites?

We did not want to ignore the paper from Patterton’s lab because it was the first one to map nucleosomes genome-wide in *T. brucei* and the main finding of that paper claimed the existence of a well-positioned nucleosome at intergenic regions, what we though constitute a point worth to be discussed. While Patterton’s work use MNase-seq from gel-purified samples and provides replicated experiments sequenced in really good depth; Siegel’s lab uses MNase-ChIP-seq of histone H3 but performs only one experiment and its input was not sequenced. So, each work has its own caveats and provides different information that together contributes to make a more comprehensive study. We think that bringing up both data sets to the discussion, as we have done in Figures 1 and 3, helps us and the community working in the field to enrich the discussion.

If the authors re- analyse the data, they should compare their pipeline to those used in the other studies, highlighting differences and potential improvements.

We are working on this point. We will provide a more detail description in the final revision.

Since many figures resemble those in already published studies, there seems little reason to repeat and compare without a detailed comparison of the pipelines and their differences.

Following the reviewer advice, we are now working on highlighting the main differences that justify analyzing the data the way we did and will be added in the finally revised method section.

At a first glance, some of the figures might look similar when looking at the original manuscripts comparing with ours. However, with a careful and detailed reading of our manuscripts you can notice that we have added several analyses that allow to unveil information that was not disclosed before.

First, we perform a systematic comparison analyzing every data set the same way from beginning to end, being the main difference with previous studies the thorough and precise prediction of TAS for the three organisms. Second, we represent the average chromatin organization relative to those predicted TASs for TriTryps and discuss their global patterns. Third, by representing the average chromatin into heatmaps, we show for the very first time, that those average nucleosome landscape are not just an average, they keep a similar organization in most of the genome. These was not done in any of the previous manuscripts except for our own (Beati, PLOS One 2023). Additionally, we introduce the discussion of how the extension of MNase reaction can affect the output of these experiments and we show 2D-plots and length distribution heatmaps to discuss this point (a point completely ignored in all the chromatin literature for trypanosomes). Furthermore, we made a far-reaching analysis by considering the contributions of each publish work even when addressed by different techniques. Finally, we discuss our findings in the context of a topic of current interest in the field, such as TriTryp’s genome compartmentalization.

Several previous Mnase- seq analysis studies addressing chromatin accessibility emphasised the importance of using varying degrees of chromatin digestion, from low to high digestion (30496478, 38959309, 27151365).

The reviewer is correct, and this point is exactly what we intended to illustrate in figure number 2. We appreciate he/she suggests these references that we are now citing in the final discussion. Just to clarify, using varying degrees of chromatin digestion is useful to make conclusions about a given organism but when comparing samples, strains, histone marks, etc. It is extremely important to do it upon selection of similar digested samples.

No information on the extent of DNA hydrolysis is provided in the original Mnase- seq studies. This key information can not be inferred from the length distribution of the sequenced reads.

The reviewer is correct that “No information on the extent of DNA hydrolysis is provided in the original Mnase-seq studies” and this is another reason why our analysis is so important to be published and discussed by the scientific community working in trypanosomes. We disagree with the reviewer in the second statement, since the level of digestion of a sequenced sample is tested by representing the length distribution of the total DNA sequenced. It is true that before sequencing you can, and should, check the level of digestion of the purified samples in an agarose gel and/or in a bioanalyzer. It could be also tested after library preparation, but before sequencing, expecting to observe the samples sizes incremented in size by the addition of the library adapters. But the final test of success when working with MNase digested samples is to analyze length of DNA molecules by representing the histograms with length distribution of the sequenced DNA molecules. Remarkably, on occasions different samples might look very similar when run in a gel, but they render different length distribution histograms and this is because the nucleosome core could be intact but they might have suffered a differential trimming of the linker DNA associated to it or even be chewed inside (see Cole Hope 2011, section 5.2, doi: 10.1016/B978-0-12-391938-0.00006-9, for a detailed explanation).

As the input material are selected, in part gel- purified mono- nucleosomal DNA bands. Furthermore, the datasets are not directly comparable, as some use native MNase, while others employ MNase after crosslinking; some involve short digestion times at 37 {degree sign} C, while others involve longer digestion at lower temperatures. Combining these datasets to support the idea of an MNase- sensitive complex at the TAS of T. brucei therefore may not be appropriate, and additional experiments using consistent methodologies would strengthen the study's conclusions.

In my opinion, describing an MNase- sensitive complex based solely on these data is not feasible. It requires specifically designed experiments using a consistent method and well- defined MNase digestion kinetics.

As the reviewer suggests, the ideal experiment would be to perform a time course of MNase reaction with all the samples in parallel, or to work with a fix time point adding increasing amounts of MNase. However, the information obtained from the detail analysis of the length distribution histogram of sequenced DNA molecules the best test of the real outcome. In fact, those samples with different digestion levels were probably not generated on purpose.

The only data sets that were gel purified are those from Mareé 2017 (Patterton’s lab), used in Figures 1, S1 and S2 and those from L. major shown in Fig 1. It was a common practice during those years, then we learned that is not necessary to gel purify, since we can sort fragment sizes later in silico when needed.

As we explained to reviewer #1, to avoid this conflict, we decided to remove this data from figures 2 and S3. In summary, the 3 remaining samples comes from the same lab, and belong to the same publication (Mareé 2022). These sample are the inputs of native MNase-ChIp-seq, obtain the same way, totally comparable among each other.

Reviewer #3 (Significance (Required)): 

Due to the lack of controlled MNase digestion, use of heterogeneous datasets, and absence of benchmarking against previous studies, the conclusions regarding MNase-sensitive complexes and their functional significance remain speculative. With standardized MNase digestion and clearly annotated datasets, this study could provide a valuable contribution to understanding chromatin regulation in TriTryps parasites.

As we have explained in the previous point our conclusions are valid since we do not compare in any figure samples coming from different treatments. The only exception to this comment could be in figure 3 when talking about MNase-ChIP-seq. We have now added a clear and explicit comment in the section and the discussion that despite having subtle differences in experimental procedures we arrive to the same results. This is the case for *T. cruzi* IP, run from crosslinked chromatin, compared to *T. brucei’s* IP, run from native chromatin.

Along the years it was observed in the chromatin field that nucleosomes are so tightly bound to DNA that crosslinking is not necessary. However, it is still a common practice specially when performing IPs. In our own hands, we did not observe any difference at the global level neither in *T. cruzi* (unpublished) nor in my previous work with yeast (compared nucleosome organization from crosslinked chromatin MNAse-seq inputs Chereji, Mol Cell, 2017 doi:10.1016/j.molcel.2016.12.009 and native MNase-seq from Ocampo, NAR, 2016 doi: 10.1093/nar/gkw068).

## 4. Description of analyses that authors prefer not to carry out

*Please include a point-by-point response explaining why some of the requested data or additional analyses might not be necessary or cannot be provided within the scope of a revision. This can be due to time or resource limitations or in case of disagreement about the necessity of such additional data given the scope of the study. Please leave empty if not applicable.*

**TAS prediction**
* Why were TAS predictions derived only from insect-stage RNA-seq data? Restricting TAS calls to one life stage risks biasing predictions toward transcripts that are highly expressed in that stage and may reduce annotation accuracy for lowly expressed or stage-specific genes. Please justify this choice and, if possible, evaluate TAS robustness using additional transcriptomes or explicitly state the limitation.

TAS predictions derived only from insect-stage RNA-seq data because in a previous study it was shown that there are no significant differences between stages in the 5’UTR processing in *T. cruzi* life stages (https://doi.org/10.3389/fgene.2020.00166). We add here a figure illustrating this point:

We are not testing an additional transcriptome here, because the robustness of the software was already probed in the original article were UTRme was described (Radio S, 2018 doi:10.3389/fgene.2018.00671).

* Please include T. brucei RNA-seq data in Suppl. Fig. S5b as you did for T. cruzi.

We have shown chromatin organization for *T. brucei* in previous S5b to illustrate that there is a similar trend. Unfortunately, we did not get a robust list of multi-copy genes for *T. brucei* as we did get for *T. cruzi,* therefore we do not want to over conclude showing the RNA-seq for these subsets of genes. The limitation is related to the fact that UTRme restrict the search and is extremely strict when calling sites at repetitive regions. Additionally, attending to the request of one reviewer we have now changed the UTR predictions for *T. brucei* using a different RNA-seq data set from Lister 427(detail in method section). Given that with the new predictions it was even harder to obtain the list of multicopy genes for T. brucei, we decided to remove that figure in the updated version of the manuscript.

* Discuss how low or absent expression of multigene families affects TAS annotation (which relies on RNA-seq) and whether annotation inaccuracies could bias the observed chromatin differences.

The mapping of occurrence and annotations that belong to repetitive regions has great complexity. UTRme is specially designed to avoid overcalling those sites. In other words, there is a chance that we could be underestimating the TASs at multicopy genes.
